# Levosimendan increases brain tissue oxygen levels after cardiopulmonary resuscitation independent of cardiac function and cerebral perfusion

**DOI:** 10.1038/s41598-021-93621-x

**Published:** 2021-07-09

**Authors:** Andreas García-Bardon, Jens Kamuf, Alexander Ziebart, Tanghua Liu, Nadia Krebs, Bastian Dünges, Robert F. Kelm, Svenja Morsbach, Kristin Mohr, Axel Heimann, Erik K. Hartmann, Serge C. Thal

**Affiliations:** 1 Department of Anesthesiology, Medical Center, Johannes Gutenberg-University, Langenbeckstraße 1, 55131 Mainz, Germany; 2 Max Planck Institute for Polymer Research, Ackermannweg 10, 55128 Mainz, Germany; 3 Institute for Neurosurgical Pathophysiology, Medical Center, Johannes Gutenberg-University, Mainz, Germany; 4 Department of Anesthesiology, HELIOS University Hospital Wuppertal, Witten/Herdecke University, Heusnerstraße 40, 42283 Wuppertal, Germany

**Keywords:** Heart failure, Neuro-vascular interactions, Brain injuries, Cerebrovascular disorders

## Abstract

Prompt reperfusion is important to rescue ischemic tissue; however, the process itself presents a key pathomechanism that contributes to a poor outcome following cardiac arrest. Experimental data have suggested the use of levosimendan to limit ischemia–reperfusion injury by improving cerebral microcirculation. However, recent studies have questioned this effect. The present study aimed to investigate the influence on hemodynamic parameters, cerebral perfusion and oxygenation following cardiac arrest by ventricular fibrillation in juvenile male pigs. Following the return of spontaneous circulation (ROSC), animals were randomly assigned to levosimendan (12 µg/kg, followed by 0.3 µg/kg/min) or vehicle treatment for 6 h. Levosimendan-treated animals showed significantly higher brain PbtO_2_ levels. This effect was not accompanied by changes in cardiac output, preload and afterload, arterial blood pressure, or cerebral microcirculation indicating a local effect. Cerebral oxygenation is key to minimizing damage, and thus, current concepts are aimed at improving impaired cardiac output or cerebral perfusion. In the present study, we showed that NIRS does not reliably detect low PbtO_2_ levels and that levosimendan increases brain oxygen content. Thus, levosimendan may present a promising therapeutic approach to rescue brain tissue at risk following cardiac arrest or ischemic events such as stroke or traumatic brain injury.

## Introduction

Despite the increased number of successful resuscitations following cardiac arrest (CA) over the last decade, survival and discharge rates as well as long-term outcomes have not improved substantially^1^. Hence, this discrepancy between successful resuscitation and patient outcome requires more effective and novel therapeutic strategies to achieve good neurocognitive function, which is the ultimate goal.

Levosimendan is an inodilator and calcium (Ca^2+^) sensitizer that was clinically established for the treatment of acute heart failure^2^. Because of its combination of inotropic and vasoactive characteristics, levosimendan has become a promising agent for post-CA care. Potential benefits during cardiopulmonary resuscitation (CPR) have been addressed in several studies, which have reported increased rates of return of spontaneous circulation (ROSC)^3^ and reduced rates of neuronal injury^4^ and organ ischemia/reperfusion injury^5^. These effects have been attributed to the known mechanisms of action of Ca^2+^ sensitization and activation of ATP-sensitive K^+^ channels in the vascular bed and activation of mitochondrial ATP-sensitive K^+^ channels in cardiomyocytes^6^. These regulations result in positive inotropy and peripheral and coronary vasodilation.

In isolated hippocampal mouse brain slices subjected to mechanical trauma, levosimendan was shown to reduce tissue injury^7^. Moreover, after 40 min of aortic clamping and consecutive spinal cord ischemia, levosimendan-treated rabbits showed better neurologic outcomes^8^. These results indicate a neuroprotective effect of levosimendan that cannot be attributed to cardiovascular changes but instead suggests a direct cellular effect on damaged tissue.

The causal link between myocardial stimulation and the observed protection following CA has not yet been determined. This study aimed to examine the significance of the cardiovascular system for levosimendan-mediated protection in a porcine model of CA by targeting the effects of levosimendan on global cerebral perfusion, cerebral microcirculation, and systemic hemodynamic parameters and correlating these changes with brain tissue oxygen levels and cerebral oxygen saturation.

## Results

### Experimental setting

ROSC was achieved in 16 of 19 animals, and all animals with ROSC survived the observation period. Before CPR, the experimental groups did not differ in terms of MAP, arterial and central venous oxygenation, lactate, total norepinephrine dose, or total amount of fluid infusion. The total length of ischemia and time from VF induction to ROSC were similar across both groups (LEVO: 674 ± 24 s, VEH: 668 ± 18 s). In all 16 animals, ROSC was achieved after a total dose of 0.8 U/kg vasopressin and a second defibrillation (360 J).

### Blood pressure and heart rate

**Figure 1:**
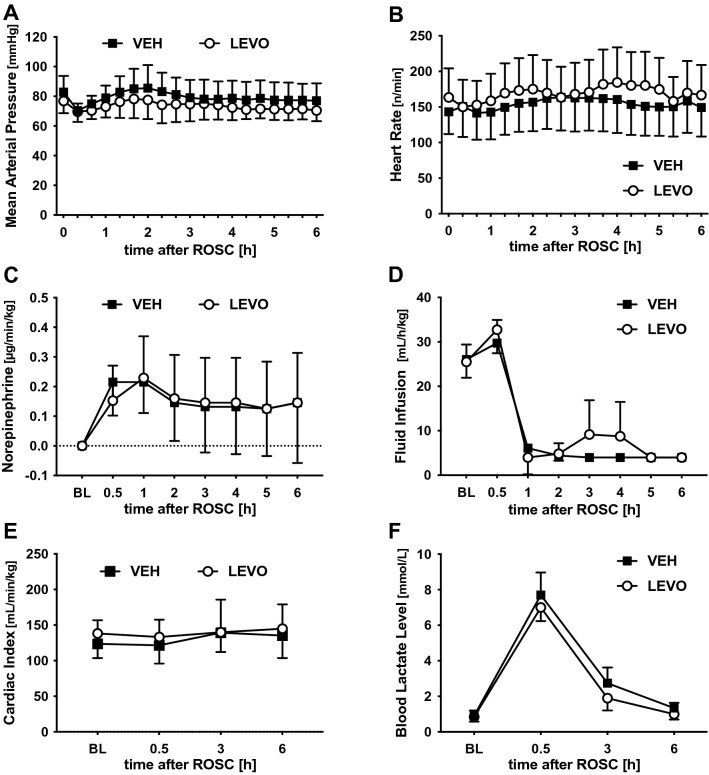
Cardiac index (CI), mean arterial pressure (MAP), and key experimental parameters. To determine the influence of cardiac arrest and levosimendan on key cardiovascular parameters and post-CPR management, MAP (**A**) and heart rate (**B**) were recorded before and after ROSC. MAP was maintained with (**C**) norepinephrine and (**D**) balanced electrolyte solution infusion. (**E**) As a general marker for cardiac function, CI was determined before and 0.5, 3, and 6 h after ROSC using the PiCCO System. (**F**) As a general marker for tissue hypoxia, blood lactate was quantified at the same time points. Levosimendan did not influence any parameter compared with vehicle treatment. *BL* baseline, *VEH* vehicle, *LEVO* levosimendan. Statistics: repeated measures two-way ANOVA, followed by Šidák’s multiple comparisons test; data are presented as means ± SD.

MAP and heart rate were recorded throughout the experiments and did not differ between the two groups (Fig. 1A,B). Similarly, vasopressor dose and balanced electrolyte solution volume required to maintain target MAP did not significantly differ between the two groups (fluid balance: LEVO: 33 mL/kg, VEH: 30 mL/kg; norepinephrine dose: LEVO: 0.15 µg/kg/min, VEH: 0.22 µg/kg/min) (Fig. 1C,D).

### Cardiac index

To determine the influence of levosimendan on post-CPR myocardial dysfunction, CI was quantified using the thermo-dilution technique and normalized to body weight. As expected, in animals without preexisting cardiovascular pathology, CI was only temporarily impaired immediately after ROSC and recovered within 30 min to prearrest baseline values (LEVO: 138 mL/min/kg; VEH: 124 mL/min/kg; Fig. 1E). Moreover, there was no significant difference between the two groups.

### Influence on serum lactate

As a marker of global tissue ischemia, serum lactate increased from normal baseline values noticeably, 30 min after ROSC (LEVO: 0.9–7.7 mmol/L; VEH: 0.85–7.0 mmol/L; Fig. 1F). In the further course, lactate returned to baseline levels within 6 h and did not differ significantly between the two groups.

### Global parameters of cardiac preload and afterload

**Figure 2:**
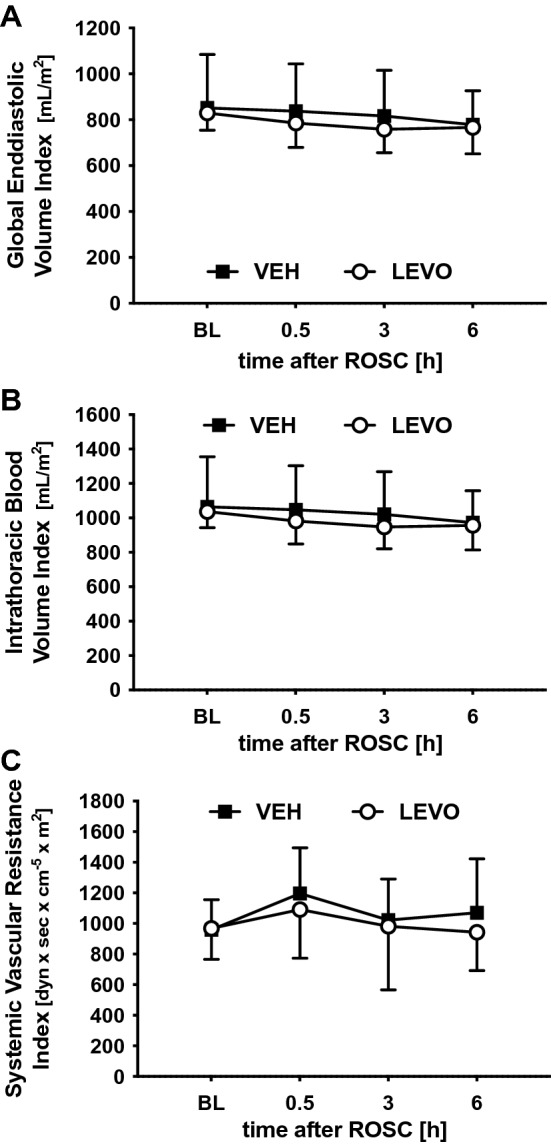
Surrogate parameters for cardiac preload and afterload. (**A**) Global end-diastolic volume index (GEDI) and (**B**) intrathoracic blood volume index as surrogate parameters for cardiac preload were determined using the PiCCO System before and 0.5, 3, and 6 h after ROSC to investigate changes in diastolic volume filling and diastolic function after cardiac arrest (CA) and levosimendan treatment. (**C**) To determine the influence on cardiac afterload, systemic vascular resistance index (SVRI) was recorded. CA and levosimendan did not influence any parameter at the selected time points. *BL* baseline, *VEH* vehicle, *LEVO* levosimendan. Statistics: repeated measures two-way ANOVA, followed by Šidák’s multiple comparisons test; data are presented as means ± SD.

GEDI (Fig. 2A) and ITBI (Fig. 2B) describe changes in volume status at the end of the diastole and the estimated blood volume in the thorax, respectively, and were used as parameters for cardiac preload. SVRI was used as a parameter for cardiac afterload (Fig. 2C) to describe the influence of levosimendan on vascular resistance. None of the three parameters were significantly influenced by treatment.

### Cerebral perfusion

**Figure 3:**
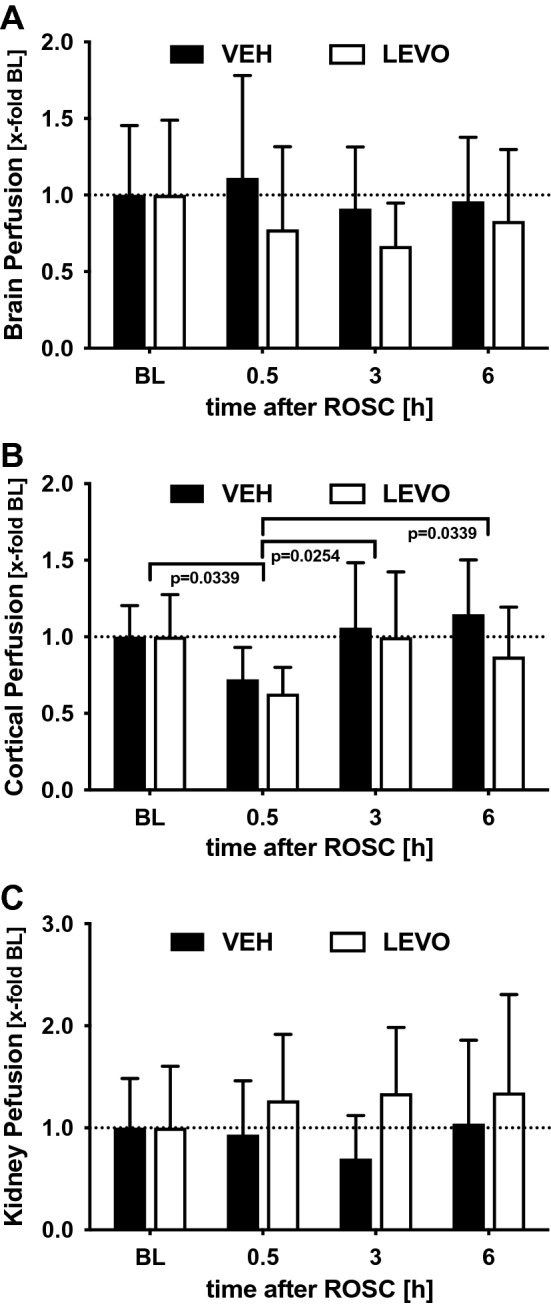
Cerebral and kidney perfusion. (**A**) Global cerebral blood flow was determined before and 0.5, 3, and 6 h after ROSC using fluorescent microspheres, and values were normalized to baseline values to correct for interindividual variation. (**B**) Cortical blood flow was measured with an epidural laser Doppler probe. (**C**) To show systemic perfusion, the kidney was selected as the index organ, and renal blood flow was measured using fluorescent microspheres. Although global brain and renal perfusion were not influenced by cardiac arrest (CA) or levosimendan treatment, brain cortical perfusion was significantly impaired 30 min after CA and returned to baseline levels at later time points. *BL* baseline, *VEH* vehicle, *LEVO* levosimendan. Statistics: repeated measures two-way ANOVA, followed by Šidák’s multiple comparisons test; data are presented as means ± SD.

To determine changes in cerebral perfusion, we used two independent techniques before and 30 min, 3 h, and 6 h after ROSC: global cerebral blood flow (CBF) was measured on the basis of fluorescent microspheres (Fig. 3A), and brain cortical blood flow was determined via O2C (Fig. 3B). To compare cerebral perfusion with perfusion in other organ systems, at all time points, total blood flow in the kidney was defined as the index (Fig. 3C). To correct for interindividual variation, all parameters were normalized to the individual baseline level obtained before CA. Total cerebral perfusion (Fig. 3A) was stable during the experiments and was not influenced by levosimendan. In contrast to total cerebral perfusion data, regional cortical blood flow (Fig. 3B) decreased significantly to 72% of baseline for VEH and 63% of baseline for LEVO following ROSC and returned to baseline values at 3 and 6 h after ROSC. In contrast to cortical cerebral perfusion, and similarly to total brain perfusion, kidney perfusion did not differ between LEVO and VEH treatment groups (Fig. 3C).

### Cerebral oxygenation

**Figure 4:**
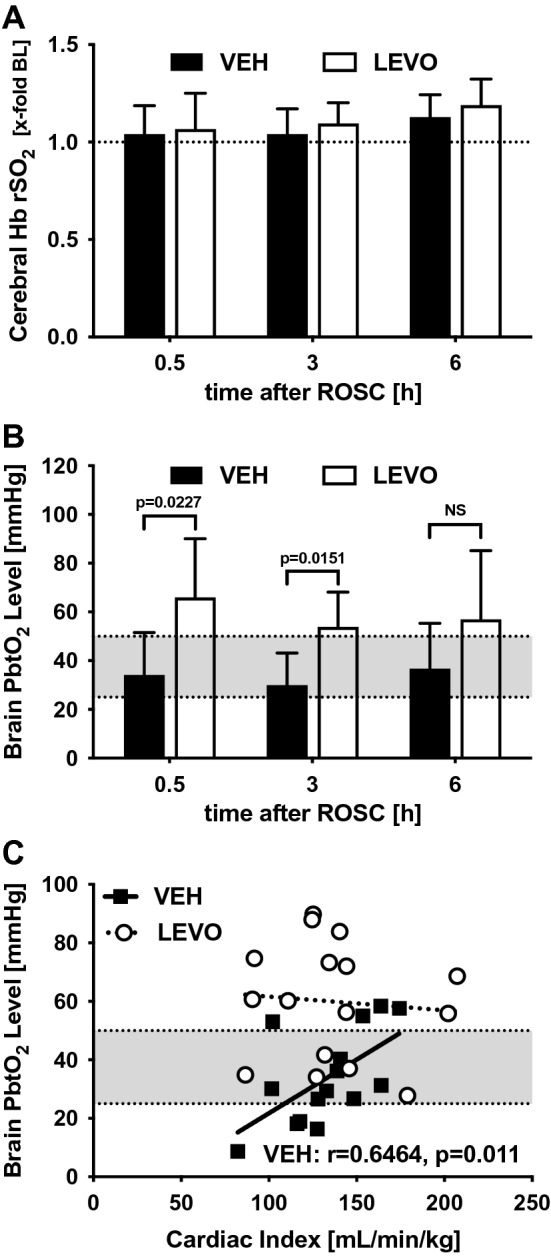
Cerebral tissue oxygenation. (**A**) Brain oxygenation was determined noninvasively by quantifying brain hemoglobin (Hb) oxygen saturation (rSO_2_) before and 0.5, 3, and 6 h after ROSC. Because of high interindividual differences, readings were normalized to baseline. rSO_2_ was not altered following cardiac arrest and did not differ between groups. *VEH* vehicle; *LEVO* levosimendan. Statistics: repeated measures two-way ANOVA, followed by Šidák’s multiple comparisons test; data are presented as means ± SD. (**B**) Brain tissue oxygen (PbtO_2_) was measured using a Foxy probe 14 mm below the dura in the left hemisphere 0.5, 3, and 6 h after ROSC. Levosimendan-treated animals showed significantly higher PbtO_2_ 30 min and 3 h after ROSC. The normal PbtO_2_ range (25–50 mmHg) is illustrated by the gray area. Statistics: Welch’s t test; data are presented as means ± SD. (**C**) Correlation analysis between PbtO_2_ and cardiac index (CI) showed a significant association between cardiac function and PbtO_2_ in vehicle-treated animals, whereas levosimendan-treated animals had normal PbtO_2_ and showed no correlation between PbtO_2_ and CI. The normal PbtO_2_ range (25–50 mmHg) is illustrated by the gray area. Statistics: Spearman rank correlation coefficient.

To identify changes in cerebral oxygenation, we used two independent techniques. We measured cortical rSO_2_ using NIRS (Fig. 4A) and PbtO_2_ in the frontal cortex using real-time O_2_ fluorescence quenching (Fig. 4B). To avoid injury to the dura mater before CA and CPR, and to prevent mechanical brain tissue injury during the CPR procedure, we did not attempt to place the brain tissue probes before CPR, and the placement of the Foxy probe was performed immediately after ROSC. To maintain optimal blood oxygenation, ventilation was adjusted as described above. Settings were confirmed by arterial blood gas analysis. Using these settings, German landrace pigs typically show a PbtO_2_ of 39.8 mmHg as recently demonstrated by our research team^9^.

NIRS values were normalized to the individual baseline level obtained prior to CA. rSO_2_ was not altered by treatment with levosimendan (Fig. 4A). In contrast to the NIRS readings, PbtO_2_ was significantly lower 30 min after ROSC. Changes in PbtO_2_ were in line with changes in cortical perfusion 30 min after ROSC. Although cortical perfusion was not improved by levosimendan, PbtO_2_ was significantly higher in the LEVO group at 30 min (LEVO: 66.0 ± 24.1 mmHg vs. VEH: 34.1 ± 17.4 mmHg, *p* = 0.0227) and 3 h (LEVO: 53.9 ± 14.3 mmHg vs. VEH: 29.9 ± 13.2 mmHg, *p* = 0.0151) after ROSC (Fig. 4B). This levosimendan-mediated increase of PbtO_2_ was independent of CI (Fig. 4C) in the levosimendan-treated animals, which all showed normal PbtO_2_, whereas PbtO_2_ correlated with CI in the vehicle-treated animals (r = 0.6464, *p* = 0.011). Although mean PbtO_2_ was in the normal range (25–50 mmHg) for both treatment groups, some of the vehicle-treated animals showed pathologically low PbtO_2_ (Fig. 4C).

## Discussion

This is the first report to directly show that levosimendan improves cerebral oxygen levels following global cerebral ischemia–reperfusion injury without improving CI or brain tissue perfusion. After resuscitation, a complex series of events occur during reperfusion, which leads to secondary brain damage^10^. The beneficial effects of levosimendan have been the subject of numerous studies^11^ that have reported increased ROSC rates^3,12^ and reduced rates of post-resuscitation myocardial dysfunction^13^, brain injury^4^, and kidney ischemia/reperfusion injury^14^. The combination of hypothermia and levosimendan has a positive effect on cardiac function, and survival has been shown to improve following CA in the rat^15^. Such positive effects have been attributed to enhanced cardiac output^11^ or CBF^4^ as the underlying mechanism. However, in contrast to this assumption, we found that levosimendan did not improve CI, any of the hemodynamic parameters, or cortical perfusion. Our study confirms findings from a study in healthy pigs without signs of left ventricular dysfunction, which showed that levosimendan does not enhance cardiac function^16^.

There is a growing body of evidence for a neuroprotective mechanism of levosimendan that is independent of any cardio-circulatory effect. Following transient spinal ischemia, levosimendan has been shown to ameliorate neurological damage^8^ and reperfusion injury in a middle cerebral artery occlusion rat model^17^, without affecting systemic hemodynamic parameters. Moreover, in an in vitro model of traumatic brain injury, levosimendan reduces secondary tissue injury^7^.

We showed that levosimendan improves PbtO_2_ 30 min after CA, without influencing perfusion at the microcirculatory or macrocirculatory level. This uncoupling between CBF and tissue oxygenation is highly relevant because cerebral oxygenation is considered to be directly dependent on cerebral flow. Interestingly, tissue oxygenation in the buccal mucosa has also shown to be improved by levosimendan compared with norepinephrine in a rat model of septic shock, without major changes in microcirculation or general hemodynamics^18^. We are confident that our dataset is highly reliable because precise methods were used to measure tissue oxygenation^19^ and tissue perfusion, such as the gold standard fluorescence microspheres method, O2C, and NIRS^20^.

In summary, cardiac output, cerebral perfusion, and tissue oxygenation data suggested that levosimendan acts at the cellular level by improving mitochondrial function. The present results may offer the missing link between in vitro and in vivo studies. A putative mechanism of action is the activation of mitoK_ATP_^21^. Levosimendan has also been shown to interact with hydrophobic targets of respiratory chain complexes by lowering the function of the respiratory chain and possibly reducing ischemia/reperfusion injury in subsarcolemmal mitochondria^22^.

The selected methods required tissue digestion to liberate microbeads to accurately quantify perfusion. Thus, it was not possible to perform molecular or histological analyses of brain tissue. Further investigations are required to confirm the role of mitoK_ATP_ and the respiratory chain complex in the observed influence of levosimendan on brain tissue oxygenation. Additionally, healthy animals that do not show cardiovascular comorbidities that are typically observed in patients with CA of cardiac origin were used in this study. Hence, the present study largely represents the clinical situation of CA due to hypoxia or hypovolemia.

## Conclusion

Optimal cerebral oxygenation is key to minimizing neurological damage during and following CA. We showed that NIRS does not reliably detect low PbtO_2_ levels and that levosimendan improves parenchymal brain oxygen content. This effect was not accompanied by an increase in cardiac output or cerebral perfusion. Thus, our data suggest a direct levosimendan-mediated effect at the cellular level, which suggests that levosimendan presents a promising therapeutic approach for rescuing brain tissue in patients with acute and critically low tissue oxygen levels following CA, stroke, or traumatic brain injury.

## Methods

### Subjects

The study was approved by the Federal Animal Ethics Committee (Landesuntersuchungsamt Rheinland-Pfalz, Koblenz; protocol number 23 177-07/G 13-1-0103) and performed in accordance with the ARRIVE guidelines and general local and federal regulations and guidelines. We made every effort to minimize the number of animals used and their suffering. A total of 19 male pigs (body weight: 28.4 ± 3.1 kg; age: 2 months) were subjected to CA and resuscitation and were then randomized to receive levosimendan or vehicle treatment. Three animals did not achieve ROSC after the second defibrillation and were therefore excluded before randomization. All other animals showed ROSC after the second defibrillation.

### Cardiac arrest (CA)

To minimize stress during transportation from the animal facility to the laboratory all animals were sedated with an intramuscular injection of ketamine (8 mg/kg) and midazolam (0.2 mg/kg). Anesthesia was induced by intravenous injections of fentanyl (4 µg/kg) and propofol (3 mg/kg) and was maintained by continuous infusion of fentanyl (8 µg/kg^/^h) and propofol (8 mg/kg/h). A single dose of atracurium (1.5 mg/kg) was administered before endotracheal intubation. Volume-controlled ventilation (AVEA Care-Fusion, San Diego, CA) was conducted (tidal volume, 8 mL/kg; positive end-expiratory pressure 5 cmH_2_O, FiO_2_ = 0.3; inspiration-to-expiration ratio, 1:2; and variable respiration rate to achieve an end-tidal, pCO_2_ < 6 kPa). Temperature was monitored continuously using a rectal probe and maintained with a heating blanket.

Using ultrasound for guidance, five femoral vascular catheters were placed: one triple-lumen central venous catheter in the right femoral vein, a 5F introducer sheath in the right femoral artery for an arterial pressure catheter placed in the thoracic descending aorta, one introducer sheath in the left femoral vein for a pacing catheter, two introducer sheaths in the left femoral artery for a PiCCO cardiac output system (Pulsion Medical Systems, Feldkirchen, Germany) and a LV-catheter for a microsphere injection placed in the left cardiac ventricle. The position of the LV-catheter was verified via pressure curve and pressure values unique to the position in the left ventricle.

Following baseline measurements, CA was induced with a pacing catheter in the right cardiac ventricle. After ventricular fibrillation (VF), ventilation and general anesthesia were discontinued. Immediately following 7 min of VF, ventilation was started at a rate of 10 min^−1^, external chest compressions were initiated with a thumping device (LUCAS 2®, Physio-Control Inc., Lund, Sweden) at a rate of 100 min^−1^, and 0.4 U/kg of vasopressin was injected. After 2 min of persisting VF, an initial defibrillation at 200 J was performed and an additional 0.4 U/kg of vasopressin was administered. Subsequently, chest compressions were continued for 2 min, followed by a second defibrillation at 360 J. All animals included in the study achieved ROSC after the second defibrillation. ROSC was defined as a presence of heart contractions and mean arterial blood pressure (MAP) above 30 mmHg. Immediately after ROSC, 30 mL/kg of balanced electrolyte solution (Sterofundin iso, B.Braun, Melsungen, Germany) were given, anesthesia was resumed, and the animals received either 12 µg/kg of levosimendan (LEVO), followed by continuous infusion of 0.3 µg/kg/min or the equivalent fluid volume of balanced electrolyte solution (BES). Randomization and preparation of the infusion were carried out by a third party who was not involved in the experimental setting.

Following ROSC, norepinephrine infusion was started in all animals at a rate of 0.3 µg/kg/min. If MAP was below 60 mmHg at 5 min after ROSC, an additional balanced electrolyte solution bolus (15 mL/kg) was administered. Target MAP was defined as 75 ± 10 mmHg. Further norepinephrine infusion was adjusted accordingly. Balanced electrolyte solution infusion was adjusted to 5 mL/kg/h, and additional boluses were injected if the global end-diastolic volume index (GEDI) dropped below 20% of the baseline value (before CPR).

**Figure 5:**
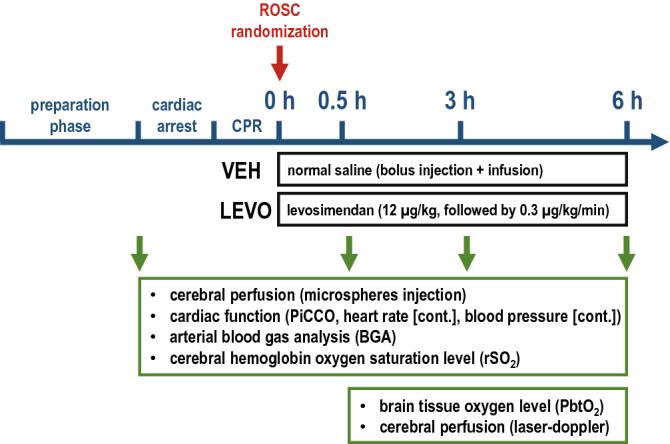
Experimental protocol.

Investigators were blind to the experimental groups. Apart from the continuous infusion of levosimendan or vehicle solution, both groups were treated identically (see Fig. 5).

### Hemodynamic variables and blood gas analysis

An arterial thermo-dilution system (PiCCO System) was used to determine and record blood pressure, cardiac index (CI), GEDI, intrathoracic blood volume index (ITBI), and systemic vascular resistance index (SVRI). All parameters were measured and recorded continuously (S5, GE Healthcare, USA). Arterial blood gas analysis was performed with a Rapidlab 248 system (Bayer Healthcare, Germany).

### Brain hemoglobin oxygen saturation

We quantified cerebral oxygen hemoglobin saturation (rSO_2_) using a near-infrared spectroscopy (NIRS) probe (Adult Soma Sensor, Covidien, Mansfield, MA, USA) placed on the right forehead. Measured values were updated and displayed in 5 s intervals using the INVOS™ 5100C cerebral/somatic oximeter (Somanetics Corporation, Troy, MI, USA).

### Cerebral tissue oxygen

PbtO_2_ content was measured using an ultrafast fiber-optic, aluminum-jacketed fluorescence-quenching pO_2_ probe with an uncoated ruthenium complex at the tip (Foxy-AL300, Ocean Optics, Dunedin, FL)^19^.

A craniotomy (1 × 1 cm) was performed on the left hemisphere, 5 mm apart from the midline and 5 mm behind the coronal suture, to allow insertion of the probe (diameter 0.5 mm) through and 14 mm below the dura.

### Laser Doppler flowmetry

Cerebral microcirculation was measured using the laser Doppler flowmetry (O2C) system (LEA Medizintechnik, Gießen, Germany). Based on the Doppler principle and light spectroscopy, the system measures regional blood flow 8 mm below the surface. The probe was positioned 1 cm lateral to the Foxy-AL300 probe on the intact dura.

### Cerebral perfusion by microspheres

The colored microsphere technique was used to measure regional blood flow as previously described^23,24^. In brief, fluorescent-labeled microspheres (15 µm diameter) were injected via a catheter into the left cardiac ventricle to be distributed throughout the body and trapped in capillaries. Four different colors were used in a randomized sequence at baseline (BL, before CPR) and 30 min, 3 h, and 6 h after ROSC (1 mL equaling 10^6^ spheres). At the end of the observation period animals were euthanized, brains and kidneys removed and separated into small samples to facilitate recovery of the microspheres by digesting the samples. The number of microspheres were determined via high-performance liquid chromatography for a highly sensitive quantification of the fluorescence levels. They were injected via a catheter into the left ventricle to be distributed throughout the body and trapped in capillaries. Four different colors were used in a randomized sequence. At baseline (BL, before CPR) and 30 min, 3 h, and 6 h after ROSC, 1 mL (equaling 10^6^ spheres) was injected. After the animals were euthanized, the complete brain and kidneys were dissected, and the microspheres were recovered.

### Statistical analysis

All experiments were performed after randomization, and the experiments and analyses were performed by investigators who were all blind to group allocation. The Prism 9 statistical software (GraphPad, La Jolla, CA, USA) was used to perform statistical analysis. Before the analysis, we assessed the test assumptions. Because of the limited power of small samples, we did not perform formal goodness-of-fit tests before the t test or analysis of variance (ANOVA); instead, we relied on the graphical assessment of distribution characteristics. Normality was determined by inspecting the unimodality and symmetry of histograms as well as by Q–Q plots. The equality of variances was determined by inspecting the histograms and standard deviations (SDs). To evaluate group differences in repeated measurements from the same animals, a repeated measures two-way ANOVA was applied (factors: treatment and time), which was followed by a Šidák multiple comparisons test. Comparisons between two independent groups were conducted using Welch’s t test. A *p* value of < 0.05 was considered statistically significant. Correlation analysis between PbtO_2_ and CI was performed using the Spearman rank correlation coefficient. Data are presented as means and SDs (means ± SD).

### Ethics approval and consent to participate

Experiments were approved by the Federal Animal Care Committee (Landesuntersuchungsamt Rheinland-Pfalz, protocol number 23 177-07/G 13-1-0103). The experiments were performed in accordance with the ARRIVE guidelines and general local and federal regulations and guidelines.

### Consent for publication

The manuscript does not include details, images, or videos relating to an individual person.

## Data Availability

All datasets generated and analyzed during this study are kept in the Dept of Anesthesiology, Medical Center of the Johannes Gutenberg-University and are available from the corresponding author upon reasonable request.

## Abbreviations

BL: Baseline
CA: Cardiac arrest
CBF: Cerebral blood flow
CI: Cardiac index
CPR: Cardiopulmonary resuscitation
GEDI: Global end-diastolic volume index
ITBI: Intrathoracic blood volume index
LEVO: Levosimendan
MAP: Mean arterial blood pressure
NIRS: Near-infrared spectroscopy
PbtO_2_: Brain tissue oxygen
ROSC: Return of spontaneous circulation
rSO_2_: Cerebral oxygen hemoglobin saturation
SVRI: Systemic vascular resistance index
VEH: Vehicle solution
VF: Ventricular fibrillation
